# High-Performance Lithium-Sulfur Batteries With an IPA/AC Modified Separator

**DOI:** 10.3389/fchem.2018.00222

**Published:** 2018-06-14

**Authors:** Yafang Guo, Aihua Jiang, Zengren Tao, Zhiyun Yang, Yaping Zeng, Jianrong Xiao

**Affiliations:** ^1^College of Science, Guilin University of Technology, Guilin, China; ^2^Guangxi Key Laboratory of Electrochemical and Magnetochemical Functional Materials, Guilin University of Technology, Guilin, China

**Keywords:** separator, active carbon, isopropyl alcohol, polysulfide adsorption, lithium-sulfur battery

## Abstract

To inhibit the polysulfide-diffusion in lithium sulfur (Li-S) batteries and improve the electrochemical properties, the commercial polypropylene (PP) was decorated by an active carbon (AC) coating with lots of electronegative oxygenic functional group of –OH. Owing to the strong adsorption of AC and the electrostatic repulsion between the –OH and negatively charged polysulfide ions, the Li-S batteries demonstrated a high initial discharge capacity of 1,656 mAh g^−1^ (approximately 99% utilization of sulfur) and the capacity can still remain at 830 mAh g^−1^ after 100 cycles at 0.2 C. Moreover, when the rate was increased to 1 C, the batteries could also possess a discharge capacity of 1,143 mAh g^−1^. The encouraging cycling stability make clear that this facile approach can successfully restrain the shuttle effect of polysulfides and make further progress to the practical application of Li-S batteries.

## Introduction

In order to meet the ever increasing demand for high-capacity, long cycle life and stable rechargeable batteries, more and more electrochemical workers are starting to pay attention to lithium sulfur batteries, which possess a high theoretical capacity (1,675 mAh g^−1^) and high specific energy (2,600 Wh kg^−1^) (Zhang et al., 2015; Zhou et al., 2015). Compared with the conventional Li-ion battery, Li-S cell displays more advantages such as cost-effective, rich reserve and environment-friendly (Zu and Manthiram, 2013; Wang et al., 2014, 2016a,b; Gong et al., 2016; Zhang et al., 2018). Nevertheless, some intrinsic properties still hindered the massive implementation of Li-S cells: (1) poor electric and ionic conductivity of S_8_ and its final reaction products (Li_2_S_2_/Li_2_S), (2) severe diffusion of the polysulfide intermediates (Li_2_S_x_, 4≤ × ≤8), (3) low electrochemical utilization of the active materials (Cai et al., 2015; Lai et al., 2015; Wang et al., 2015; Zhu et al., 2016).

Tremendous efforts have been devoted to solve these scientific issues in Li-S cells by holding sulfur in various composites with special structures or exploiting new electrolytes. Although significant progress has been made in the utilization of elemental sulfur and the cyclic stability, the synthetic methods are usually relatively complex, which not only need a variety of additives but also have a higher requirements for the manufacturing processes (Xiong et al., 2012; Huang et al., 2013; Wang et al., 2013; Hu et al., 2014; Zhang et al., 2014; Deng et al., 2015; Lee et al., 2015; Liu et al., 2016; Lu et al., 2016; Nersisyan et al., 2016; Yang et al., 2016). Alternatively, modifying the commercial separators have been proved to be a facile and commendable strategy to improve the electrochemical performance through effective regulation of polysulfide shuttle (Chung and Manthiram, 2014a,b; Li G. C. et al., 2015; Balach et al., 2016; Conder et al., 2016; Fan et al., 2016). Especially the introduction of functional groups on the surface of separator is gradually studied. For instance, Yu X et al. modified the separator with carboxyl functional group through a sequence of hydroxylating, grafting and hydrolyzing processes to bring about a negatively charged environment in Li-S cells (Yu et al., 2016). In order to constrain the diffusion of electronegative polysulfides, Li Z et al. introduced oxygenic functional groups (-OH, -COOH) onto the surface of separator by O_2_ plasma treatment (Li Z. et al., 2015). Similarly, a method of one-step plasma-induced graft co-polymerisation was used to develop negatively charged –${\text{SO}}_{3}^{-}$ onto the microporous membrane and this separator showed a good ability to inhibit the shuttle effect (Conder et al., 2015).

In this study, we present a facile approach to achieve a high-performance active carbon coated separator with hydroxyl groups, which can perform excellent physical adsorption and electrostatic exclusion at the same time, bringing about a strong inhibition of soluble electronegative polysulfides. In comparison to the batteries assembled using pristine PP separator, the Li-S batteries with modified separator exhibit significantly enhanced cyclic stability and rate capability.

## Experimental section

### Materials preparation

First, 1.0 g active carbon was added to 30 mL isopropyl alcohol (IPA) and magnetic stirred for 24 h to permeate IPA into the pores of active carbon. Then the prepared solution was dried at 60°C for 6 h to obtain IPA/AC composite material.

A slurry method was used to coat the PP (Celgard 2400) separator with the IPA/AC composite. A mixture of IPA/AC composite and polyvinylidene fluoride (PVDF) (8:1, by mass) was placed in N-methy-2-pyrolidone (NMP) to form slurry, which was subsequently coated on the cathode side of the pristine PP separator. The IPA/AC modified separator was then dried in vacuum oven at 60°C for 4 h. In addition, AC modified separator was prepared in the same way for comparison.

### Material characterization

The morphology was characterized by a field emission scanning electron microscopy (SEM, HTTAHIS-4800). Energy dispersive spectrometer (EDS) was employed to identify the distribution of the elements on the surface of the IPA/AC-coated separators. The chemical state of the carbon and oxygen in samples were tested with X-ray photoelectron spectroscopy (XPS, ESCA LAB 250Xi).

### Battery assembly and electrochemical measurement

A solid solution method was used to fabricate the active composite materials with a mixture of S_8_ and AC (7:3, by mass). Sulfur cathode was made of as-prepared S/AC composite, acetylene black and PVDF dissolved in NMP with a mass ratio of 7:2:1. The obtained homogeneous slurry was coated onto aluminum foil with a doctor blade, followed by drying in a vacuum oven at 60°C for 12 h. The active substance sulfur loading was about 3.17 mg cm^−2^.

CR-2025-type button cells were assembled in an argon-filled glove box with pristine separators, AC-coated separators and IPA/AC modified separators for comparison. Lithium metal was used as the counter electrode. The electrolyte consisted of 1.0 wt% LiNO_3_ and 1.0 M LiTFSI in a mixed solvent of DME and DOL at the volume ratio of 1:1.

Cyclic voltammetry (CV) were measured by a CHI750E electrochemical workstation at a scan rate of 0.1 mV s^−1^ within the voltage range of 1.5–3.0 V. Electrochemical impedance spectroscopy (EIS) of the cells was carried in the frequency range of 10 mHz−100 kHz with a perturbation amplitude of 5 mV. In addition, galvanostatic charge-discharge tests and rate capability were conducted to evaluate the cycle stability of Li-S cells on the basic of S_8_ at different current densities under LAND test instrument.

## Results and discussion

### Characterization of IPA/AC modified separators and pristine separators

**Scheme 1:**
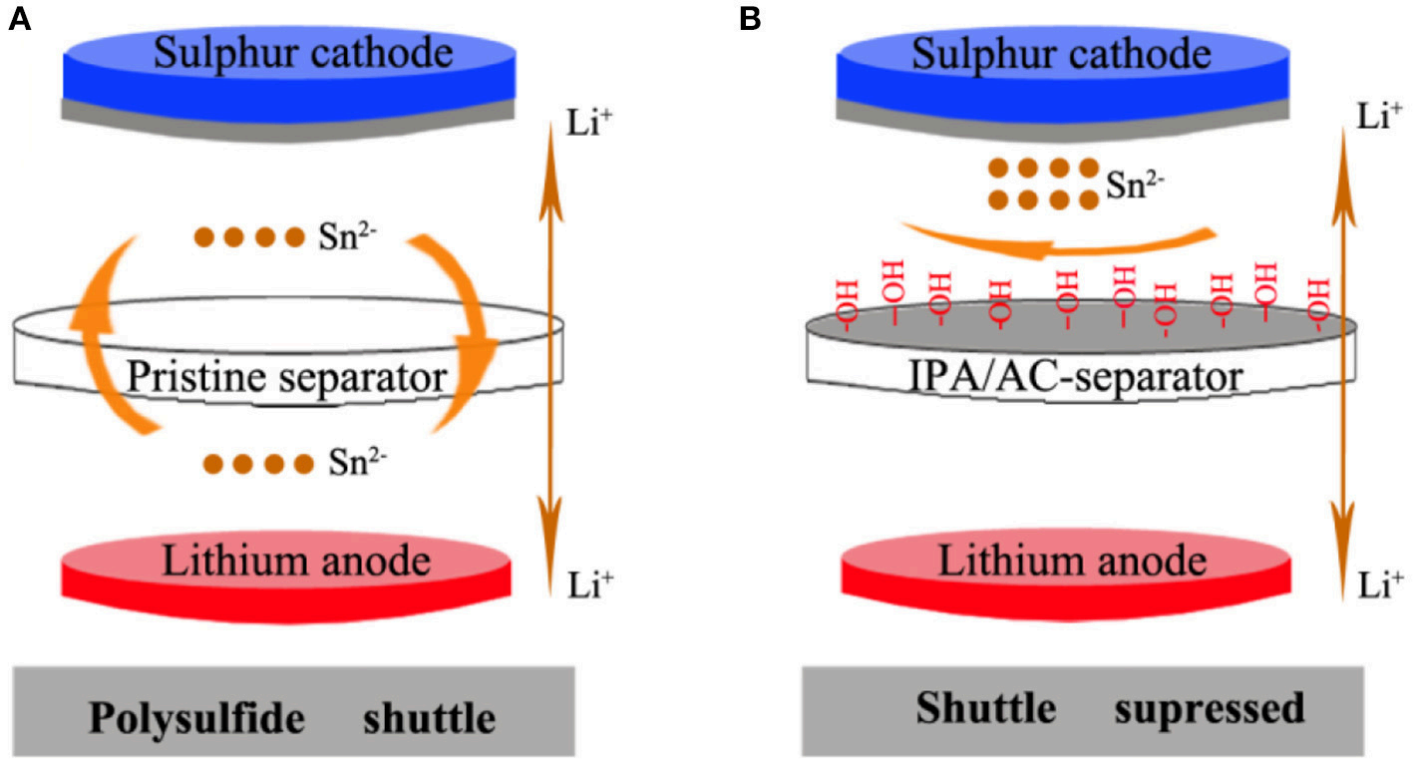
**(A)** Polysulfide diffusion in Li-S cells with pristine separator and **(B)** Inhibition of polysulfide-diffusion in Li-S cells with the IPA/AC modified separator.

The shuttling effect inhibition principle in Li-S cell is showed in Scheme 1. In the battery with pristine separator, the polysulfide ions of Sn^2−^ (4≤ n ≤8) can freely shuttle back and forth between the two poles. But the shuttle effect can be effectively suppressed in the battery with IPA/AC modified separator. From Scheme 1B we can see the IPA/AC coating is on one side of the bare separator, facing the sulfur cathode and act as a surface barrier. This barrier contains porous active carbon with strong adsorption and electronegtive oxygenic functional group of –OH, which can simultaneously take advantage of physical adsorption and electrostatic repulsion to prevent the diffusion of polysulfide ions to the lithium anode.

**Figure 1:**
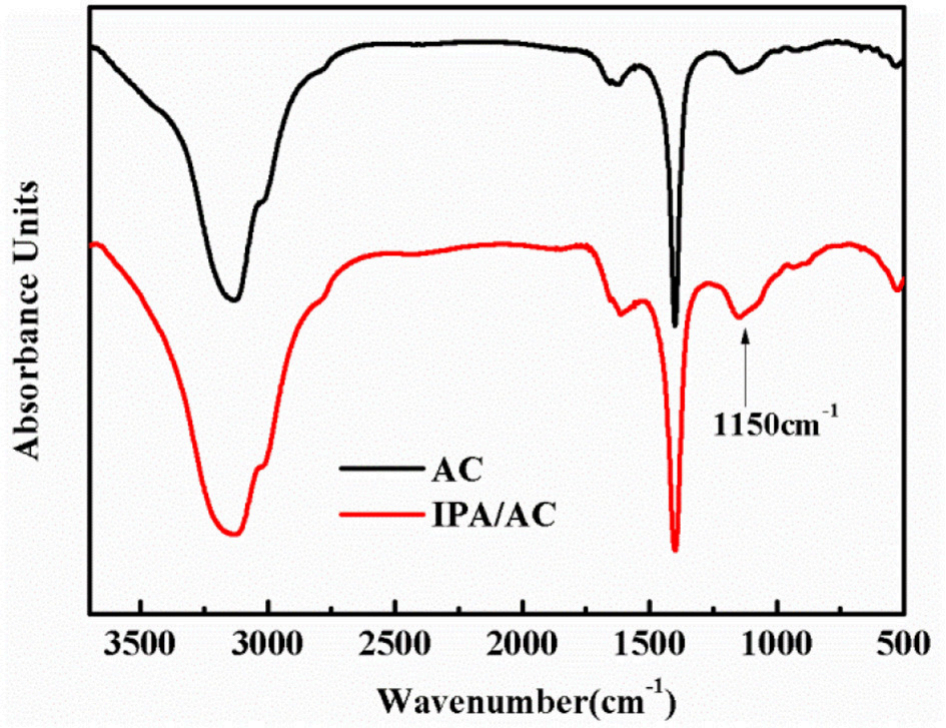
FTIR spectra of AC and IPA/AC.

To investigate the functional groups that exist in IPA/AC composites, we performed the FTIR characterizations on the samples. Figure 1 gives the FTIR spectrum of AC and IPA/AC. The wide peak in the FTIR spectrum of IPA/AC at 1,150 cm^−1^ is the characteristic peak of –OH, by comparing the intensity and width of the peaks, we can confirm that hydroxyl groups are successfully introduced into the IPA/AC composites.

**Figure 2:**
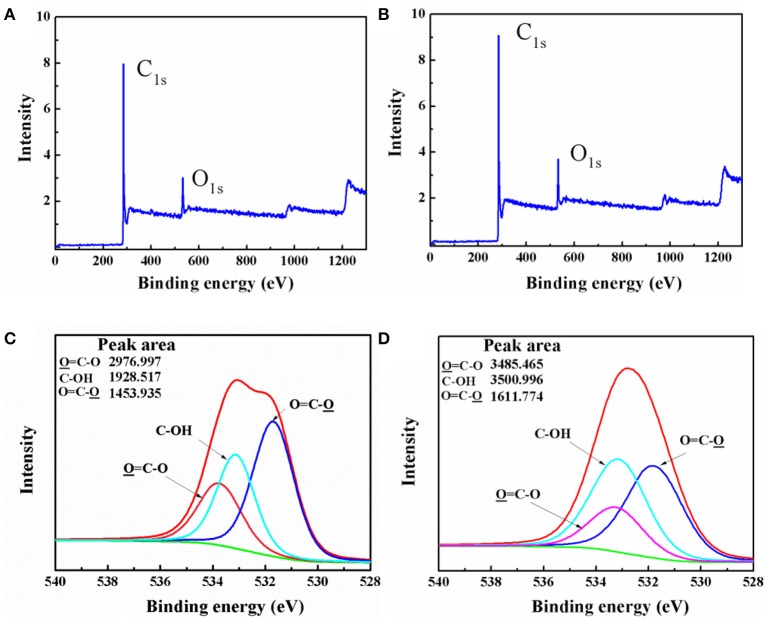
XPS survey spectrum of **(A)** AC and **(B)** IPA/AC. High-resolution O1s XPS spectra of **(C)** AC and **(D)** IPA/AC.

The content of C1s and O1s in active carbon materials and IPA/AC composites were measured by X-ray photoelectron spectroscopy (XPS). Figures 2A,B show the intensity of C1s and O1s in IPA/AC (9 and 3.8, respectively) are obviously higher than those in AC (8 and 3, respectively), indicating that IPA/AC contains more C1s and O1s. High-resolution O1s XPS spectra of IPA/AC is shown in Figure 2D, three peaks can be easily identified at the binding energy 531.71, 533.08, and 533.17 eV, corresponding to O = C-O, C-OH and O = C-O groups, respectively (López et al., 1991; Stevens et al., 2014), which are similar to the spectrum of AC in Figure 2C. To determine how much C-OH was introduced, we calculated the percentage of its peak area. The result shows the C-OH in IPA/AC is 40%, higher than that of AC (30%). This consequence reveals that hydroxyl group was successfully introduced in to the active carbon particles.

**Figure 3:**
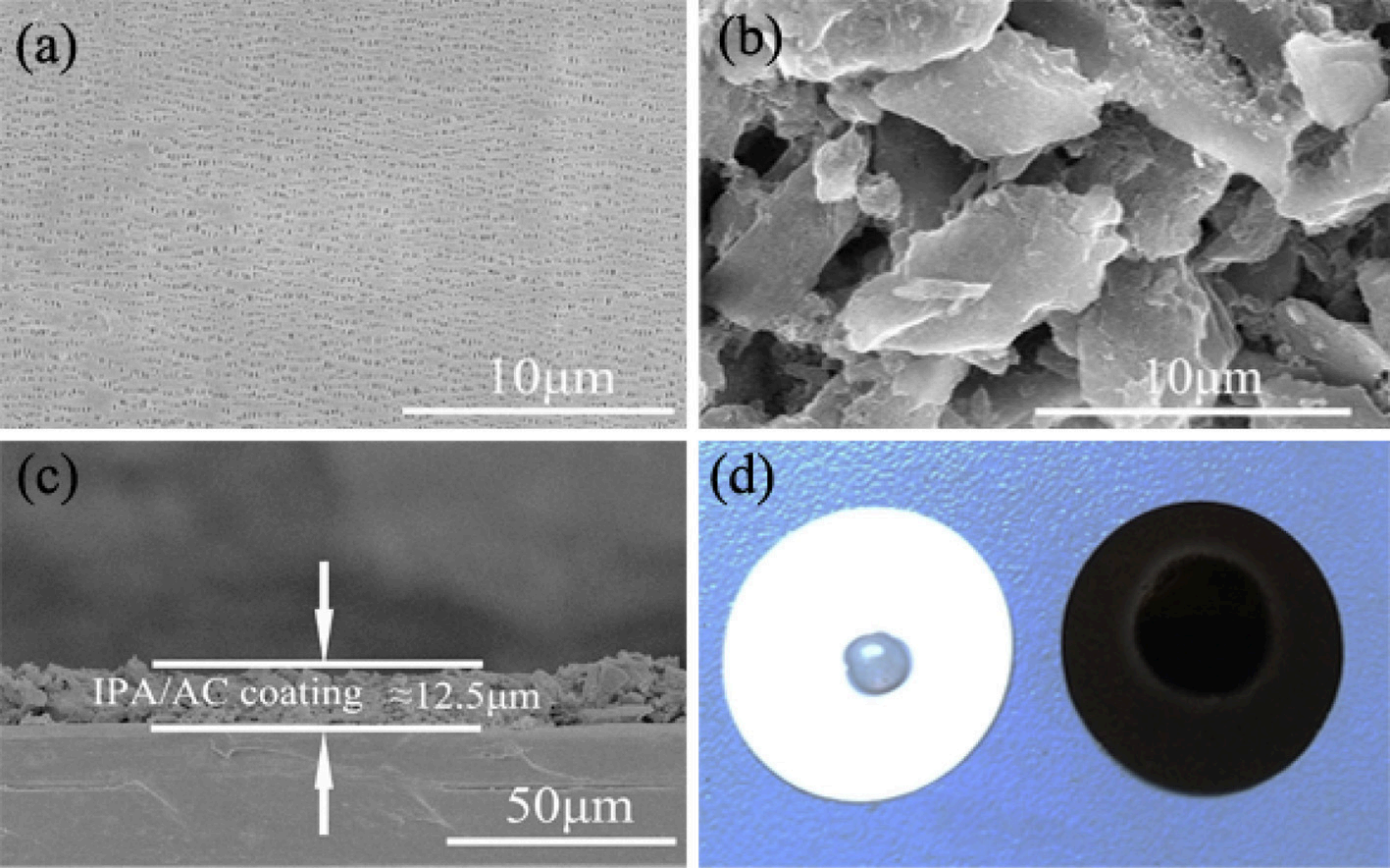
SEM images of **(a)** pristine Celgard separator and **(b)** the IPA/AC coated separator. **(c)** Cross-sectional SEM image of IPA/AC modified separator. **(d)** Wetting behavior of electrolyte on the pristine separator and IPA/AC modified separator.

SEM was used to examine the morphology of the original separator and IPA/AC modified separator, as given in Figures 3a,b. A smooth surface with uniformly substantial slit pores structure is presented for routine separator, which promotes ion conduction but restricts the transportation of electron (Gong et al., 2009). In comparison with the bare separator, the surface of IPA/AC modified separator is covered with micrometer active carbon particles. These particles have a large specific surface area and superior conductivity, which can not only provide rich attachment points for polysulfide ions but also contribute to reducing the internal resistance of Li-S battery.

Figure 3c reveals the cross-section of IPA/AC modified separator. From the image we know the IPA/AC coating is about 12.5 μm and in good contact with the Celgard separator. Figure 3d shows the electrolyte affinity test of the routine separator and IPA/AC modified separator. As seen, the droplet is not dispersed on the routine separator, whereas the IPA/AC modified separator wetted a large area. It can be predicted that the electrochemical performance of Li-S battery with IPA/AC modified separator would be significantly improved, during to an increased rate of ion transmission.

**Figure 4:**
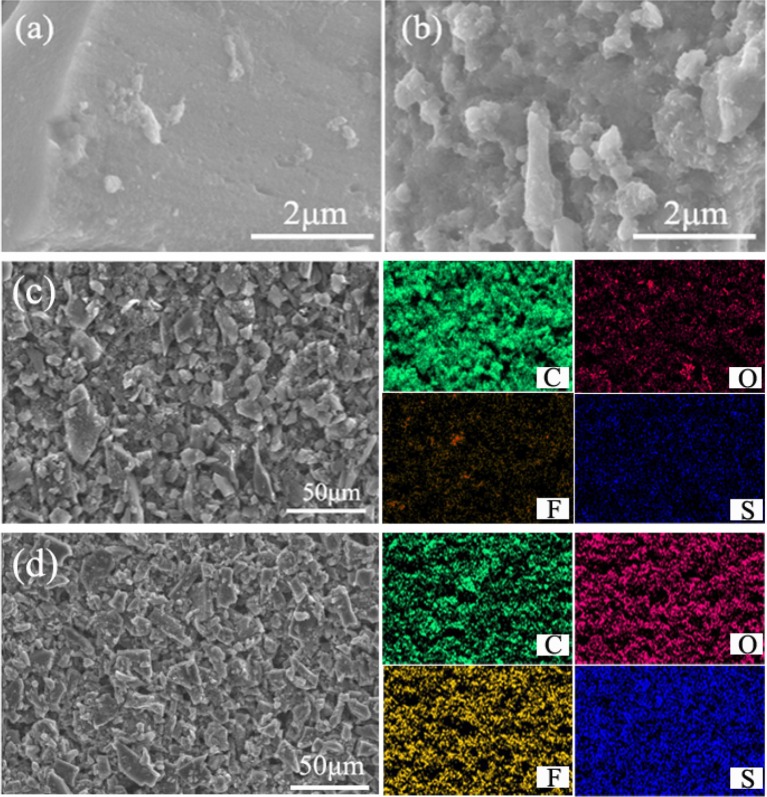
High-magnification SEM images of the IPA/AC modified separator **(a)** before cycles and **(b)** after 100 cycles at 0.2 C. SEM images and elemental mapping of the IPA/AC coated separator **(c)** before cycles and **(d)** after 100 cycles at 0.2C.

To ascertain the utility of IPA/AC coating, the morphological changes before and after cycling were observed, as summarized in Figures 4a,b. Before cycling, the surface of IPA/AC particles is relatively smooth (Figure 4a), while after 100 cycles the surface turns rough with clumps of different sizes, which indicates the effective physical adsorption and electrostatic repulsion of free dissolved polysulfides in cathode region (Figure 4b). For further supporting this conclusion, the contrast of elemental mappings before and after 100 cycles at 0.2C is conducted by energy dispersive spectrometer (EDS). As given in Figures 4c,d, only very weak elemental signals of O, F and S could be detected on the IPA/AC coating before cycling (Figure 4c), and these faint element signals may come from the impurities in active carbon materials. After cycles these signals became obvious and distributed evenly, which can be attributed to the fully infiltration of the electrolyte and the mass attachment of the polysulfides as well (Figure 4d).

### Electrochemical performance of batteries with IPA/AC modified separator

**Figure 5:**
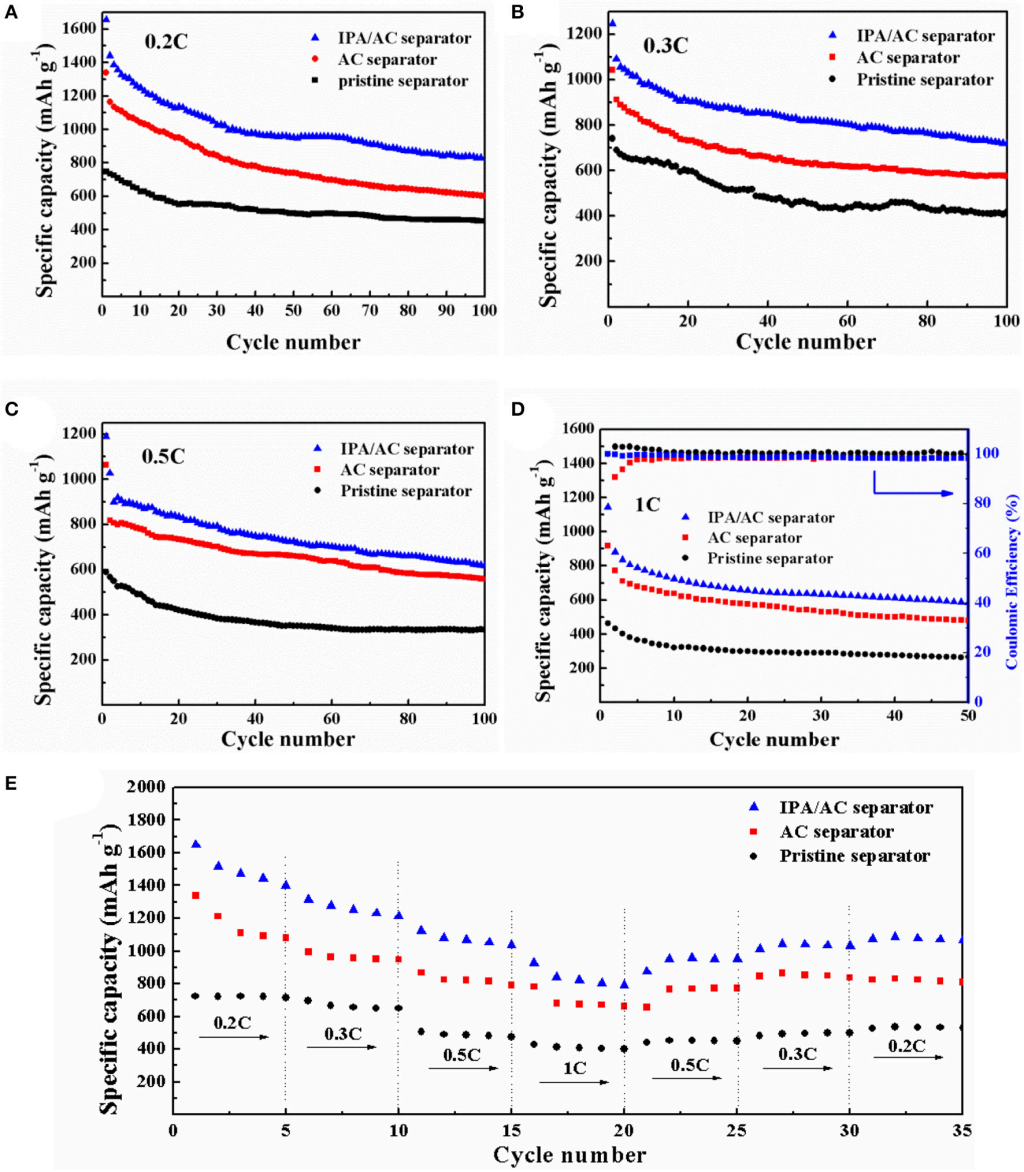
The cycling performance of Li-S cells with pristine separator, AC coated separator and IPA/AC modified separator at **(A)** 0.2 C, **(B)** 0.3 C, **(C)** 0.5 C, and **(D)** 1 C. **(E)** Rate performance of Li-S cells with pristine separator, AC-coated separator and IPA/AC modified separator.

The function of IPA/AC modified separator on electrochemical performance is investigated based on CR-2025-type button cells. Cyclic performance at different discharge current rate of Li-S batteries using IPA/AC modified separator, AC-coated separator and original separator for comparison are presented in Figures 5A–D. As anticipated, the cell with IPA/AC modified separator reveals a significant enhancement at each current rate. The initial discharge capacities of the cells with IPA/AC modified separators are 1,656, 1,246, 1,190 and 1,143 mAh g^−1^ at 0.2C, 0.3C, 0.5C, and 1C, respectively, which are much higher than those of the other two cells at these current rate, as given in Table 1. In particular, after 50 cycles at high current rate of 1C, the capacity of Li-S cell with IPA/AC modified separator can maintain at 584 mAh g^−1^, and the Coulombic efficiency is above 98%. Therefore, we can conclude that the reaction intermediates are largely trapped within the IPA/AC coating, which effectively reduces the irreversible loss of active substances.

**Table 1:** The cycling performance of Li-S cells with IPA/AC modified separators, AC-coated separators and pristine separators at different current rates (mAh g^−1^).

**Sample**	**0.2C**		**0.3C**		**0.5C**		**1C**	
	**1st**	**100th**	**1st**	**100th**	**1st**	**100th**	**1st**	**50th**
IPA/AC separator	1,656	830	1,246	719	1,190	618	1,143	584
AC separator	1,339	602	1,042	575	1,063	558	918	479
Pristine separator	747	452	741	417	589	333	462	265

Figure 5E presents the rate performance of three Li-S batteries at a vary current rate of 0.2C → 0.3C → 0.5C → 1C → 0.5C → 0.3C → 0.2C. From this chart we can see the cell with IPA/AC modified separator delivered the highest initial discharge capacity of 1,650 mAh g^−1^ at 0.2C, demonstrating the high utilization of sulfur which can be ascribed to the easy penetration of electrolyte and the significant blocking effect of the IPA/AC coating. When increased the current rate to 1C, the capacity of this battery is as high as 927 mAh g^−1^, while only 781 and 427 mAh g^−1^ could be obtained from the cells with AC-coated separator and bare separator, respectively. In addition, after 35 cycles, the capacity of IPA/AC sample still retained at 1066 mAh g^−1^ (approximately 65% of the initial reversible capacity), attesting to the efficient electrostatic repulsion between the –OH and negatively charged polysulfide ions, leading to a excellent rate performance of Li-S cell.

**Figure 6:**
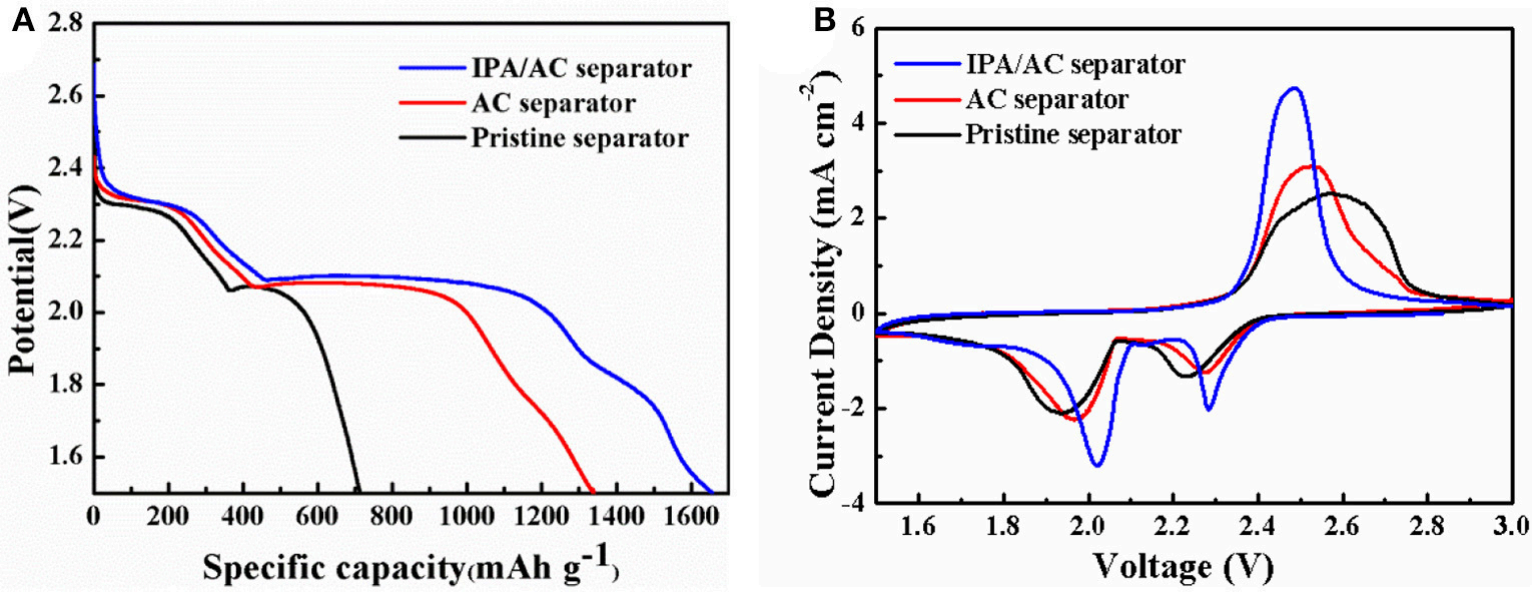
**(A)**Initial discharge profiles of Li-S cells with pristine separator, AC-coated separator and IPA/AC modified separator at 0.2 C. **(B)** Cyclic voltammetry of Li-S cells with pristine separator, AC-coated separator and IPA/AC modified separator at a 0.1 mV s^−1^ scanning rate.

The initial discharge profiles of the Li-S batteries using IPA/AC modified separator, AC-coated separator and pristine separator at 0.2 C are exhibited in Figure 6A. It is found that each profiles consists of two typical discharge potential plateaus corresponding to the reduction from elemental sulfur to long-chain polysulfides at high voltages and from long-chain polysulfides to short-chain Li_2_S_2_/Li_2_S (Jianrong et al., 2014; Guo et al., 2017). However, there are distinctly differences in the height and length of the plateau. Apparently, the IPA/AC separator battery possesses the highest and longest voltage platform, revealing the considerable utilization of active materials along with a thorough chemical reaction. Figure 6B gives the initial cyclic voltammetry curves (CV) of the three batteries at a scanning rate of 0.1 mV s^−1^. One anodic peak and two cathodic peaks can be discerned from these CV curves. And the positions of these two cathodic are consistent with the discharge potential plateaus of discharge profiles (Xiao et al., 2015). Moreover, one thing we should pay attention is that in IPA/AC battery the position of cathodic peaks are higher than those of the other two batteries, while the position of anodic peak is lower, indicating that the IPA/AC coating can not only reduce the charge voltage but also make a great improvement on the discharge depth, displaying the admirable transport kinetic of ions and electrons.

**Figure 7:**
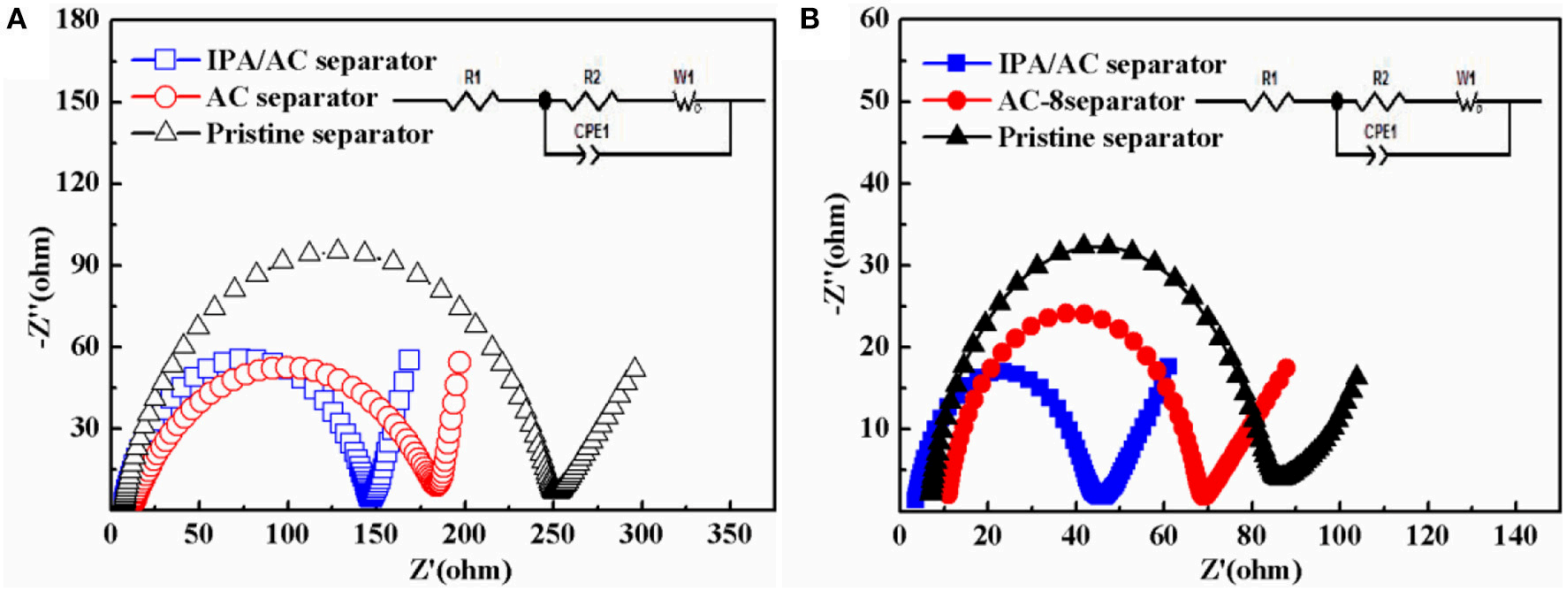
Electrochemical impedance spectrum (EIS) of the cells with pristine separator, AC-coated separator and IPA/AC modified separator **(A)** before and **(B)** after 100 cycles at 0.2 C.

The enhanced electrochemical performance is further verified by the electrochemical impedance spectrum (EIS) measurement within a frequency range of 10 mHz−100 kHz and the equivalent circuit is acquired by Z-view software. In the equivalent circuit, R_1_ denotes the resistance of the electrolyte, R_2_ is the charge transfer resistance, CPE_1_ represents the constant-phase elements, and W_1_ is the Warburg diffusion impedance (Figure 7 inset) (Hou et al., 2017). From Figure 7, a semicircle can be saw at high and medium frequency, representing the charge transfer resistance (Rct) (Li G. C. et al., 2015). From the diameter of the semicircle we know the cells with AC-coated separator and pristine separator have a larger Rct than IPA/AC sample both before and after cycles, demonstrating the reduction of the charge transfer resistance by the special function of the IPA/AC coating, which can act as a surface collector to reserve enough electrolyte and accelerate the diffusion of lithium ion.

**Figure 8:**
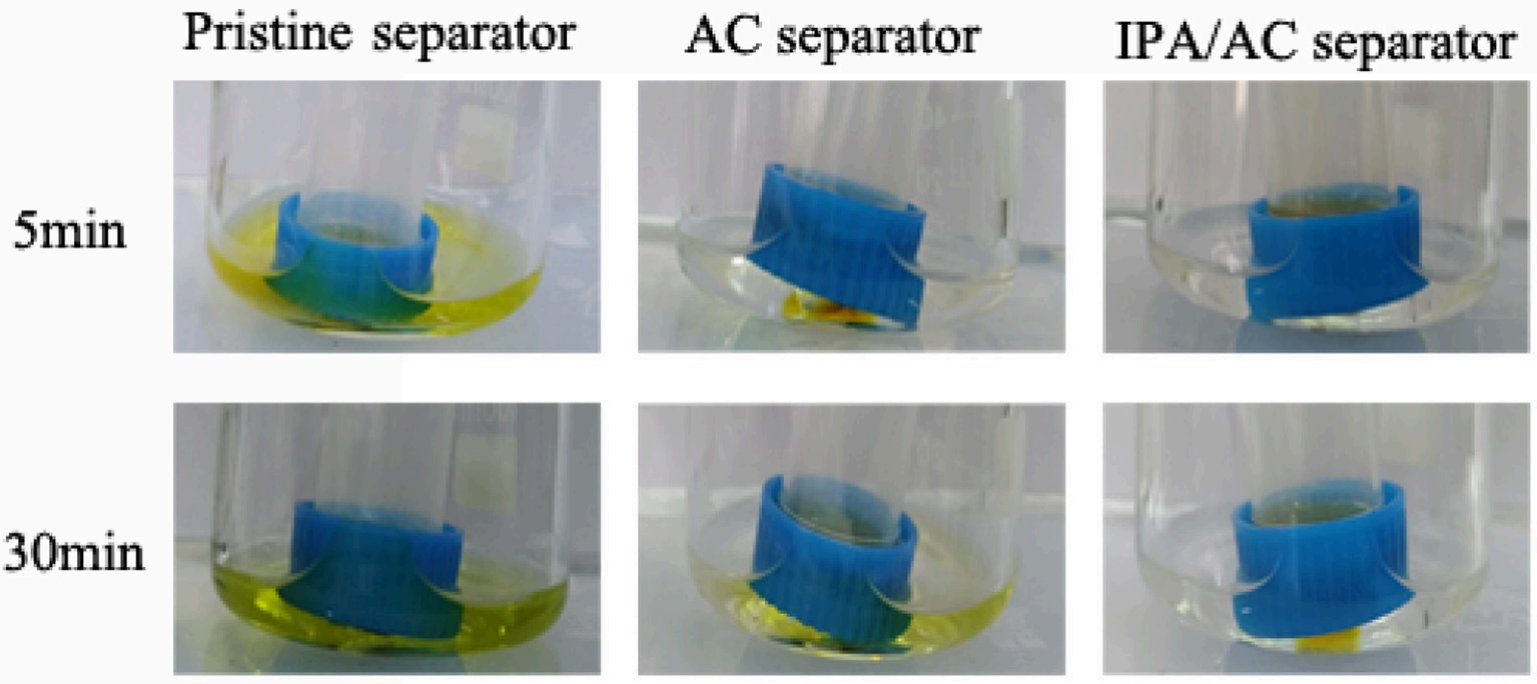
Polysulfide diffusion test of pristine separator, AC separator and IPA/AC separator.

In order to visual observe the retention of polysulfide species by the introduced IPA/AC separator, we conducted the polysulfide diffusion test for the three separator samples, as shown in Figure 8. The polysulfide solution in glass tubes was made by adding 7.77 mg S_8_ and 2.23 mg Li_2_S into 5 ml DME:DOL (1:1,v:v), and solution in beakers was 4 ml DME:DOL (1:1,v :v). As expected, the pristine separator does not suppress the diffusion of polysulfides, thus the color of DME:DOL solution already changed to yellow after 5 min of rest. In contrast, the IPA/AC separator largely suppressed the diffusion of polysulfide species, therefore even after 30 min of rest only a little change in color was observed, indicating that the porous active carbon coating with hydroxyl groups has good retention capability of polysulfide, which is attributed to the physical adsorption of porous carbon structure and the electrostatic repulsion between the hydroxyl and negatively charged polysulfide ions.

## Conclusion

In conclusion, the IPA/AC modified separator successfully integrates the strong physical adsorption of active carbon and the electrostatic repulsion between the hydroxyl and negatively charged polysulfide ions to obstruct the shuttle effect in the Li-S battery. With this special coating, the battery can present a apparent improvement on the storage of electrolyte, ion conduction and the utilization of active substances, leading to a stable cycle ability and excellent rate performance. In addition, the modification method only acquires active carbon and isopropyl alcohol, which is environment-friendly and easy to operate, indicating that the IPA/AC modified separator provides a great potential in the commercial production of lithium sulfur battery.

## Author contributions

All authors listed have made a substantial, direct and intellectual contribution to the work, and approved it for publication.

### Conflict of interest statement

The authors declare that the research was conducted in the absence of any commercial or financial relationships that could be construed as a potential conflict of interest. The reviewer, BQ, and handling Editor declared their shared affiliation.
